# Presentation and management of ACE-I induced angioedema in the Emergency Department: an observational study

**DOI:** 10.1186/1710-1492-10-S1-A23

**Published:** 2014-03-03

**Authors:** R Mason Curtis, Sarah L Felder, Rozita Borici-Mazi, Ian M Ball

**Affiliations:** 1 Department of Emergency Medicine, Queen’s University, Kingston, Ontario, Canada; 2 Division of Allergy and Immunology, Queen’s University, Kingston, Ontario, Canada; 3 Program in Critical Care Medicine, Queen’s University, Kingston, Ontario, Canada; 4 Department of Biomedical and Molecular Sciences, Queen’s University, Kingston, Ontario, Canada

## Background

Angioedema of the upper airway is a life-threatening Emergency Department (ED) presentation with many etiologies. Angiotensin converting enzyme-inhibitor (ACE-I) use is one cause of non-mast cell mediated angioedema. As the use of this medication class increases with our aging population, it is important to characterize the presentation and management of ACE-I induced angioedema (AAE), a rare but potentially severe side effect of this commonly prescribed medication class. The objectives of this study were to describe the incidence and management of AAE in the ED and to identify any epinephrine-induced morbidity.

## Methods

A retrospective medical record review was conducted of all patients presenting to two Canadian tertiary care EDs between July 2007 and March 2012. Patients were identified for inclusion using the *International Classification of Diseases*, *10^th^ revision* discharge diagnostic codes of T782, T783, T784, T886, T887 and D841. Records were excluded from study if there was no visible swelling documented on the medical record, or if swelling was found to be secondary to non-systemic reaction to an insect sting, trauma or irritant exposure.

## Results

Of 1702 medical records identified through our inclusion criteria, 1175 were excluded for reasons cited above. Of the remaining 527 ED visits, an inciting cause was identified in 48.8% (n=257), based on our *a priori* definitions. Of these, the most common identifiable etiology was AAE (33.1%, n= 85). The most common locations of swelling in patients with AAE were the tongue or lips, found in 51% and 40% of subjects, respectively. Common ED medications used to manage AAE included diphenhydramine (63.5%, n=54), corticosteroids (50.6%, n=43) and ranitidine (31.8%, n= 27). Epinephrine was administered in 18 AAE patients (21.2%), 5 of whom received repeated doses. In two of these patients, morbidity developed shortly after epinephrine administration, causing myocardial ischemia or dysrhythmia. Four AAE patients (4.7%) required admission to hospital and one required endotracheal intubation. There was no associated mortality.

## Conclusions

Our study demonstrates that AAE is the most common identifiable etiology of angioedema of patients presenting to the ED. Management of AAE commonly includes antihistamines and corticosteroids. Concerningly, epinephrine use is common. Many patients started on ACE-Is are at high risk for developing complications from epinephrine administration, which has limited physiologic rationale for use in the setting of AAE. More research is required to better delineate epinephrine’s role in this vulnerable patient population and identify other therapeutic options.

